# Pathological Specimens Presented to the Cincinnati Academy of Medicine, February 26, 1900

**Published:** 1900-04

**Authors:** Rufus B. Hall

**Affiliations:** Cincinnati, O.


### PATHOLOGICAL SPECIMENS PRESENTED TO THE CINCINNATI ACADEMY OF MEDICINE, FEBRUARY 26, 1900.

By RUFUS B. HALL, A.M., M.D.,
CINCINNATI, O.

This group of specimens occurring in my recent work is of unusual interest, each one illustrating some interesting feature in the work of abdominal surgery. The busy life of the hard worker and the great number of specimens passing through the hands of men engaged in this line of work make one hesitate to present a specimen before such a body of medical gentlemen as the Academy represents, unless some interesting feature is connected or associated with the case from which the specimen has been removed, or something unusually interesting in the specimen itself. I hope that the specimens and cases will not prove uninteresting to you even though many of you do not do the class of work in which I am engaged.

The first specimen presented this evening is a multinodular fibroid tumor of the uterus, removed from M., aged thirty-six, patient of Dr. James F. Heady, of Glendale, Ohio. The operation was made at my private hospital January 20, 1900. The patient had been conscious of the presence of the tumor for several years. She suffered from pressure and profuse menstruation during all this time but was able to enjoy ordinary health until four or five months ago. Since that time she has suffered severely from uterine hemorrhage, which was almost constantly present. The patient was almost exsanguinated. The anemia was profound. The tumor was somewhat larger than an adult head, a portion of it blocking up the pelvic cavity. To the right side of the tumor and above it was a somewhat movable mass that was in size and shape not unlike the normal kidney. For four or five weeks preceding the operation the patient suffered great pain at times, and there was a good deal of doubt as to the cause of the pain, whether or not the pressure of the tumor upon the ureter at the pelvic brim, damming back the urine in the kidney, was not the cause. It could not be made out before the operation whether this somewhat movable lump was a portion of the tumor, or the right kidney which was movable. The examination of the urine was negative. The The shape of the tumor in palpation was so like a kidney that it made the diagnosis difficult as to that feature in the case. She had a left side femoral hernia somewhat larger than the closed hand which had existed for several years, was irreducible and not strangulated. At the time of the operation when the abdomen was opened it was found that the omentum was glued down to the tumor on the left side. It was pulled well down and a large portion of the omentum constituted the hernia spoken of. The omentum was firmly adherent in the sac and could not be liberated from the inside of the abdomen. I could not make the manipulations necessary for the removal of the tumor without liberating the omentum. Therefore I ligated it close to the femoral ring at two points an inch apart and cut between the ligatures, leaving the portion comprising the hernia undisturbed. Under the tumor was an adherent hematoma of the ovary holding a small quantity of old dark blood-clot. This was ruptured during the manipulation and a small portion spilled upon the field of operation. Total extirpation was made and the operation finished in the usual way. She developed a severe attack of peritonitis. I have little doubt but that it was the spilling of the hematoma that caused this. After a severe struggle of three days convalescence was established and she made a nice recovery. At the commencement of the operation I expected to remove the tumor and repair the hernia at the same sitting, but she took the anesthetic so badly and her condition was so desperate by the time the operation for removal of the tumor was completed that it precluded any further operative interference. I was greatly interested and a little worried to know what would become of the large mass of omentum filling the hernia sac. I hoped that the adhesions to the sac would be sufficient to sustain its vitality. If so all would be well. If not, I reasoned that a secondary operation a few days hence by laying the sac open and treating it as an open wound would give her a better chance for recovery than to prolong the operation at this time. I expected this hernia to give her trouble within a few days, and I was prepared to meet it when it came and to do whatever was necessary to be done. To my great delight there was never any tenderness, swelling, redness or undue tension in the hernia sac. At the end of two weeks it commenced to diminish in size, and when she left the hospital, five weeks after the day of her operation, it was but very little larger than a hulled black walnut.

This case emphasizes a conviction I have entertained for several years as to the danger of infection from an old hematoma of the ovary in these operations for removal of fibroid tumors. In patients where this blood is spilled over the field of operation nearly every one of them develop severe peritonitis, and we all appreciate what a dangerous risk that is to our patient. I have come to regard this hematoma to be as dangerous as pus. The practical lesson to be drawn from a case of this kind is earlier operation, before the ovaries are embraced between the pelvic walls and the tumor and form adhesions favoring the development of hematoma. The hernial condition is so rare that I do not wish to comment upon it. By examination of the tumor itself you will observe this pedunculated mass on the top of the tumor and to one side. This is exactly the shape of a normal kidney and somewhat larger. It has a pedicle an inch long and about as thick as a man's finger. You can readily understand why this might be mistaken through the thin abdominal walls for a kidney.

CASE II. The second specimen is a typical parovarian cyst holding about a gallon and a half of fluid at the time of the operation. It was removed from Mrs. D., aged 26, of Piqua, Ohio, a patient referred to me by Dr. Grosvenor. She was operated upon at the Presbyterian hospital January 24th, 1900. She has been married two years—no children. Soon after marriage she noticed that her abdomen was gradually enlarging, which continued up to the present time. For the past four or five months she had been losing flesh and strength and was much emaciated. She suffered from indigestion caused by pressure.

You will observe from this specimen that the ovary is normal in size and attached to the side of the specimen. The cyst is a beautiful illustration of a typical parovarian cyst, and I present it for that reason. The remaining ovary had a cyst on one side as large as a hen's egg. This cyst was excised, as the remaining part of the ovary appeared perfectly normal. Where the ovary was incised it was stitched over with fine catgut and left. The patient made an uninterrupted recovery and went home four weeks from the day of her operation.

CASE III. The third specimen here presented was removed from Mrs. P., aged 47, from Lynn, Ky., a patient of Dr. Morris. The patient was brought to the Presbyterian hospital on January 20th, and was quite ill when she entered that institution at noon of that day. She had a pulse of 120 and temperature 101. By evening of the same day the pulse was 130 and temperature 104. She had a tumor in the abdomen which distended the abdomen about as much as a woman at full term of pregnancy. She suffered great pain in the right half of the abdomen corresponding with the vermiform appendix. She was restless, vomited frequently, and for three days her pulse varied from 140 to 150 per minute and temperature from 102 to 104. She had regurgitant vomiting and it looked like she was going to die within a very short time. By repeated small doses of calomel and high rectal injections we succeeded on the third day in getting her bowels moved thoroughly, and in twenty-four hours afterwards convalescence was established. She was fed all the stomach would assimilate, and her general health was improved in every way possible up to February 5th, 1900, when the operation was made. This patient lived ten or twelve miles in the country from Greenup, Ky., the nearest railroad station. In September of last year she was conscious of the presence of a small tumor in her abdomen, not much larger than her closed hand. About this time she had a severe attack of pain in the right half of the abdomen, and the doctor believed that she had an attack of appendicitis in connection with her tumor. She was confined to bed for several weeks and had never regained her usual health. She was able to be up about the room, but the riding in the wagon to reach the railroad train the day she came to the hospital and the journey of 120 miles by rail, started the abdominal inflammation anew, from which she nearly lost her life.

The tumor, as you will observe, is a multilocular ovarian tumor. At the time of the operation, it was found that the tumor was adherent to the whole anterior wall of the abdomen. The vermiform appendix was adherent between the tumor and the abdominal wall. It had perforated and an abscess cavity had formed, the abdominal wall forming one side and the tumor wall the other side of this cavity. Infection had taken place in the tumor and one large cyst contained pus as part of its contents. The tumor was adherent to nothing except the abdominal wall and the head of the colon. There was a long pedicle. I removed the appendix, and you will observe that it is perforated near its tip. This patient made a nice and easy recovery.

CASE IV. This multinodular fibroid tumor was removed from Mrs. W., aged 39, of Sidney, Ohio, a patient of Dr. Costolo. She was operated on at my private hospital February 20th. This patient had suffered from pelvic and abdominal pain for several years, but a correct diagnosis of the condition was not made until recently, when she came under the charge of her present physician. He immediately advised operative interference for obvious reasons. This large, irregular mass was wedged down in her pelvis and caused so much pain from pressure that her life was made miserable and she was anxious for relief. The patient has made an easy convalescence and will recover. From her clinical history we could say with considerable confidence that she was at an end of comfortable existence with this tumor, and any delay now meant complications in the operation and greater risk when it was made.

CASE V. This specimen is the most interesting one of the group. It was removed from Mrs. L., aged 37, a resident of this city. The patient was referred to me by Dr. J. S. Caldwell and was operated upon at my private hospital on February 21st. She is the mother of two children, the younger eleven years old. She had her first acute pelvic inflammation some five years ago, which was probably due to infection. Since that time she has never been well, having recurrent attacks once or twice every year with varying severity. For a year or more her suffering has been very great. Upon examination the whole pelvic cavity was found filled full. The uterus was retroverted and fixed. It could not be lifted up or moved by any manipulation from the vagina. There was a well defined, hard mass at both sides of the uterus. She suffered several severe attacks of hemorrhage in the past few months, corresponding with her menstrual periods.

Upon opening the abdomen the intestines and omentum were found thoroughly agglutinated to the mass in the pelvis. Both tubes were occluded and contained pus. The adhesions to the intestines, omentum and pelvic floor were very firm. After liberating the coils of bowel and omentum I decided to remove both ovaries and tubes with the retroverted uterus, and I here present the specimens as a whole. If you will observe the specimen you will see that every portion that should be normal peritoneum is covered with shreds of adventitious tissue representing the adhesions. The operation was a very tedious and difficult one. I am convinced of the wisdom of making a hysterectomy in all operations for relief of old inflammatory cases where both sides must be sacrificed, especially if the woman is near the menopause. The danger from secondary hemorrhage in case of vomiting from the anesthetic is very much less because each blood-vessel is taken up and tied separately and no tension is brought upon it to cause slipping or loosening of the ligature if vomiting should occur. Again, these patients suffer less afterward from the pain corresponding with their menstrual periods during their enforced menopause, and they are saved the annoyance of the endometritis that is coexistent with their pelvic disease. They will make an earlier, smoother and more satisfactory recovery both to their physician and to themselves.

